# Breaking barriers: feasibility of a cluster randomised trial evaluating an instrument for identifying and ameliorating adverse drug reactions

**DOI:** 10.1136/bmjopen-2025-099627

**Published:** 2026-01-27

**Authors:** Vera Logan, David Hughes, Adam Turner, Neil Carter, Sue Jordan

**Affiliations:** 1 Faculty of Medicine, Health and Life Sciences, Swansea University, Swansea, UK

**Keywords:** Adverse events, Polypharmacy, Medication Review, Aged, Feasibility Studies, Primary Health Care

## Abstract

**Abstract:**

**Objectives:**

We aimed to investigate the feasibility of a cluster randomised controlled trial (RCT) of the ADRe Profile in UK primary care. The ADRe Profile is a patient monitoring system to identify and address adverse drug reactions (ADR) and ADR-related issues to pre-empt clinical deterioration.

**Design:**

A preliminary study to test the feasibility of an RCT.

**Setting:**

General practices (GPs) in South-West Wales, UK.

**Participants:**

20 patients aged >64 and prescribed >4 long-term medicines.

**Interventions:**

Participants reported their health-related problems using the ADRe-Profile. Participants completed the profile independently initially, then with researcher support, capturing vital signs, clinical observations and patient-reported symptoms.

**Main outcome measures:**

Feasibility was assessed based on recruitment, retention, adherence to protocols, potential for clinical impact and staff costs.

**Results:**

We recruited two GP practices (0.94% of 213 contacted), and 20 patients aged >64 (51.3% of those approached). Retention was 100%. ADRe Profiles had a 98.29% completion rate, identifying 289 clinical problems, including pain (16 of 20 patients), dyspnoea (10/20), dizziness (8/20), bleeding/bruising (7/20) and falls (4/20). Most problems (90%) and vital signs (78%) recorded on ADRe Profiles were absent from existing patient records. Researchers recommended further investigations (164 instances) and interventions (126 suggestions). Despite the potential for clinical benefits, engagement from clinicians was limited. Cost estimates for ADRe administration ranged from £40 to £73, within the funding available from Health and Care Research Wales.

**Conclusions:**

An RCT of the ADRe Profile was feasible, despite gatekeeping by clinicians. Recruitment of GP practices was challenging, with <1% of eligible practices participating. In contrast, patient recruitment and retention were successful. ADRe aligns with WHO patient safety goals and could improve healthcare by addressing ADR-related problems proactively in this vulnerable population.

**Trial registration number:**

NCT04663360; Pre-Results.

**Strengths and limitations of this study**

- This comprehensive exploration of the feasibility of a randomised controlled trial identified differences between clinicians and patients in attitudes towards medicines monitoring.
- The high number of previously undocumented problems, such as pain, dyspnoea, emesis, falls and serious bleeding, highlights an unmet clinical need for preventive strategies, overlooked by busy clinicians, and a normalisation of actual and potential harm.
- Evaluation of real-life clinician behaviours was characterised by suboptimal involvement.
- Clinician recruitment challenges suggest potential barriers in the implementation of patient-centred medicines monitoring and study findings.

## Introduction

 The patient harm attributable to preventable adverse reactions to prescribed medicines has been documented for decades.^1 2^ Much of the cost of adverse drug reactions (ADRs) falls on secondary care.^3^ Admissions due to ADRs are increasing,^4^ estimated as 11% of unplanned adult admissions in Wales,^5^ but up to 16.5% in one large English hospital.^6^ Over a 1-month period, these admissions cost £490 716. Extrapolated across NHS England 2018–2019, this implies a cost of £2.21bn from a total Department of Health and Social Care budget of £164.8bn (1.3%).^7^ However, prevention largely depends on primary care,^8 9^ particularly effective review of repeat prescriptions.^10^,^12^ More avoidable harm is attributed to suboptimal monitoring than prescribing.^2 13 14^ Since no easy solution has been found, the healthcare community needs to consider the ‘difficult’, piecemeal solutions, such as improved inter-professional cooperation, better patient engagement and development of appropriate monitoring, screening and decision-support instruments.

The WHO’s policy brief on ‘medicines without harm’ asks all countries to commit to implementing action plans for polypharmacy, high-risk medicines (eg, antipsychotics), and transition of care, and promote ‘active participation of patients through education and engagement for safe medication use’ (WHO, p.36).^11^ However, current guidelines focus on single diseases or single medicines, while most older patients use several medicines.^12 14^ The few instruments designed to monitor patients for the effects of multiple medicines are not widely used, but are, in the main, effective in mitigating ADRs.^15^ The ADRe Profile is one such system that reduced clinical problems, such as pain, falls and infections, in randomised controlled trials (RCTs) in care homes and outpatients^16 17^ and observational studies in community mental health and care homes.^918^,^21^ Uptake of freely available, person-centred patient safety medication monitoring systems is far from universal, in part because of professional ‘gatekeeping’.

Evidence from pragmatic clinical trials is essential to convince stakeholders of the value of interventions.^22^ However, many clinical trials fail to recruit,^23 24^ complete or report,^25^ despite considerable expenditure, thus generating ‘research waste’ and no social benefits.^26 27^ To pre-empt these problems, feasibility studies in target clinical areas are recommended as a precursor to full-scale trials.^28 29^ These test practical aspects of research designs, address logistical challenges, identify potential issues, refine methods and minimise risks and threats to validity,^30^,^34^ while assessing the acceptability of implementation in the real world. Exposition of this type of preparatory work is rarely described in the methodological literature,^35^ and the present paper is intended to share our approach and experience.

Conducting clinical trials in primary care has become increasingly difficult.^36^ UK primary care services report increasing workloads and staff shortages,^37^,^41^ and clinicians fear monitoring patients for ADRs will increase workload when resources are already stretched.^15^ This investigation sought to evaluate the feasibility and acceptability of the ADRe Profile in general practice (GP) surgeries for older adults prescribed multiple medicines. Before initiating the RCT, a non-randomised feasibility study was conducted to identify and address logistical challenges. Accordingly, we assessed: (1) recruitment and retention, (2) protocol modifications, (3) completion rates for the ADRe Profile, and patterns of missing items in electronic and paper formats, (4) overlap and alignment with data in GP records, (5) potential benefits and clinical gains and (6) estimated costs of implementing the ADRe Profile.

The work is registered with the ClinicalTrials.gov as NCT04663360 (https://clinicaltrials.gov/study/NCT04663360).

## Methods

This was a single-arm feasibility study to assess the practicalities of implementing a research intervention in real-world practice.^30^,^33^ Reporting was guided by the Consolidated Standards of Reporting Trials extension to randomised pilot and feasibility trials (online supplemental file 1),^29^ guidelines for reporting non-randomised pilot and feasibility studies^42^ and the Template for Intervention Description and Replication (TIDieR) checklist (online supplemental file 2).^43^

### Patient and public involvement

The Swansea University Patient Experience and Evaluation in Research (PEER) group was consulted on the research proposal and reviewed the Participant Information Sheet. PEER members provided positive feedback on the study’s purpose and offered suggestions, for example, simplification of the Participant Information Sheet. Two members of PEER joined the steering group.

### Setting

The study was undertaken in primary care in Wales, UK. The Gross Domestic Product (GDP) per capita of Wales is £27 274/$35 183,^44^ lower than the UK as a whole and below most developed countries.^45^

### Recruitment

Between November 2020 and August 2021, we approached all 213 GPs in four NHS Health Boards in South West Wales (contactable via https://primarycareone.nhs.wales/cluster-working/find-a-cluster/) with a request for research collaboration, supported by offer of reimbursement for staff costs from Health and Care Research Wales (HCRW). HCRW advertised the study on their website. Invitation letters were emailed to 114 potential participants, including GP cluster leads, general practitioners, pharmacists, nurse practitioners and PRIME Centre Wales. Pharmacists based in GP practices who expressed interest approached GPs and practice managers.

### Participants

A formal sample size calculation was not undertaken as this was a non-randomised feasibility study^30^; our sample size was justified, using two process criteria.^46^ From experience with other Adverse Drug Reaction Profile (ADRe) studies, we expected a patient participation rate above 60%, and 60%–100% to report a potential ADR that could be ameliorated.^916^,^21^ To ascertain a prevalence within 95% CIs of 60% to 100% 15 participants were needed.^47^ In each collaborating practice, patients’ usual clinician (a clinical pharmacist) accessed the practice database and selected and approached suitable participants. Table 1 lists inclusion criteria.

**Table 1:** Participant inclusion criteria

Criterion	Eligibility
Age	65 years or older
Comorbidities	1 or more long-term condition
Number of prescribed medicines	5 or more regular long-term prescriptions (vitamin and nutritional supplements and moisturising skin preparations were not counted as medicines)
If lacking capacity	A consultee or representative available

### Study procedures

**Figure 1:**
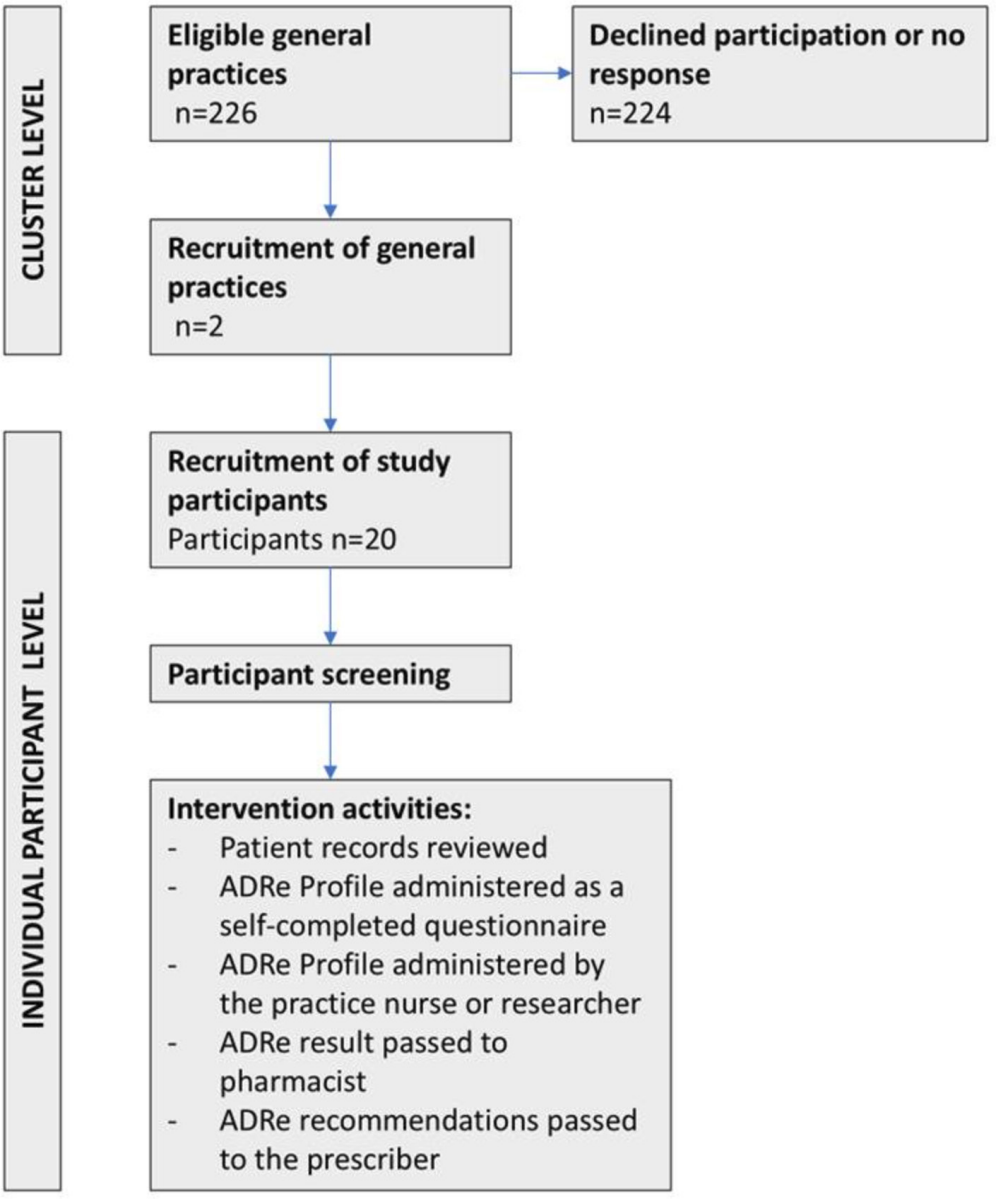
Study flow chart. ADRe, adverse drug reaction.

Practice pharmacists sent contact details of potential participants to the research team. Participants were contacted by researchers and written consent was obtained between 1 July 2021 and 15 March 2022. Figure 1 illustrates study activity.

Researchers reviewed participants’ primary care (GP) patient records (including nursing and medical notes) to identify any signs and symptoms relevant to populating the ADRe Profile. Lists of prescribed medicines were extracted.

Due to COVID-19 precautions, the ADRe Profile was administered remotely using telephone, internet or post. The ADRe Profile was posted to all participants, with a stamped addressed envelope, for self-completion and return. In the event of non-completion, a telephone reminder, followed by a postal reminder, was sent to the participants. When self-completed ADRe Profiles were returned, the researcher completed a second, identical ADRe Profile with each participant via either a videocall or a telephone call. Data from the two completed ADRe Profiles (self-completed and researcher-completed) and patient records were compared. The completed ADRe Profile, along with the list of medicines, was then forwarded to the practice pharmacist for review. Recommendations from the academic team were passed to the GP/prescriber. Researchers planned to discuss potential changes in clinical or process outcomes with the pharmacist or GP.

### Outcome measures

Feasibility criteria were determined by pragmatism and potential clinical value (table 2).

**Table 2:** Feasibility test objectives and outcome measures

Objective	Measurement
Pragmatic criteria	
(1) To assess the feasibility of recruitment and retention of GP practices and individual participants.	Recruitment and retention of practices and patients.
(2) To report adherence and fidelity to study procedures, and the need for protocol modification.	Completeness and timeframes of study procedures, protocol modifications.
(3) To investigate fidelity and completion rate of the ADRe Profile.	Number and nature of items completed/not completed independently by the patient. Number and nature of items completed/not completed in a videocall consultation. Items with a high proportion of missing data.
Clinical criteria	
(4) To establish information overlap and congruence between ADRe and patient records.	Proportion and nature of items on the ADRe Profile that can be populated from primary care patient records.
(5) To explore preliminary data on the clinical impact of the ADRe profile.	New problems identified (number and nature) not in primary care records. Estimate of associations with prescriptions and potential to improve clinical outcomes.
(6) To ascertain whether the potential staff costs of implementing ADRe would be within the research support offered by Health and Care Research Wales: £56 per participant for the feasibility study and £112 for the RCT.	Personal Social Services Research Unit unit costs of health workers in relation to reimbursement.

No proportions were pre-specified.

Field notes were taken to collect relevant contextual data.^81^

ADRe, adverse drug reaction; RCT, randomised controlled trial.

Staff costs were described using the Personal Social Services Research Unit (PSSRU) unit costs of health workers (www.pssru.ac.uk) and the English National Tariff Payment System (https://www.england.nhs.uk/pay-syst/national-tariff/national-tariff-payment-system/).

### Progression criteria

Progression was predicated on feasibility, clinical relevance and staff costs, using established criteria.^48^ The decision involved adjustments needed to mitigate risks or deficiencies (table 3).

**Table 3:** Progression criteria

Step	Criterion	Task
Assess recruitment.	Recruitment of at least two general practices and 20 patients by end of July 2021.	Verify the target was met. If not, identify barriers and consider actions.
Analyse eligibility and consent rates.	At least 40% of identified patients meet eligibility criteria, and at least 40% of these consent to participate.	Review data. If below threshold, investigate and implement strategies to improve recruitment or revise eligibility criteria.
Evaluate participant enrolment rate.	Participants should be enrolled at a rate of no less than 30% of the target sample per month.	Determine the enrolment rate. If too slow, analyse delays and plan corrective measures.
Assess ADRe Profile completion.	At least 50% of patients should self-complete the ADRe Profile (excluding vital signs).	Evaluate self-completion rate. If below the threshold, identify barriers to self-completion and modify instructions.
Evaluate the identification of ADRs.	The ADRe Profile should identify potential ADRs in at least 60% of participants.	Analyse data to confirm ADR detection rates. If below threshold, investigate and review the ADRe Profile.
Conduct staff cost approximation.	The study should be affordable, in line with practice reimbursement.	Check alignment with reimbursement.

ADRe, adverse drug reaction.

### Data analysis

Descriptive statistics were calculated for demographic data and ADRe items. Completion rate for all ADRe items was calculated for self-completed ADRe and researcher-completed ADRe. Information overlap between patient records and the ADRe Profile was calculated. Number and nature of ADRe-identified problems and suggestions for improvement were tabulated. Analyses were undertaken using the Statistical Package for the Social Sciences (SPSS, V.30).

Potential risks and benefits to participants were considered. Based on previous safe use of the ADRe Profile in care homes and community mental health teams,^9 16 18 19 21^ combined with the notion that the ADRe Profile aims to enhance patient monitoring to inform professional judgement, and that all questions and observations are of a nature that should be pursued under routine care, the potential benefits of the study were thought to outweigh any risks arising from completing the ADRe Profile. Any changes emanating from the study intervention were instigated by the patients’ usual healthcare providers/professionals and were not mandated by the research project.

## Results

### Recruitment and retention

**Figure 2:**
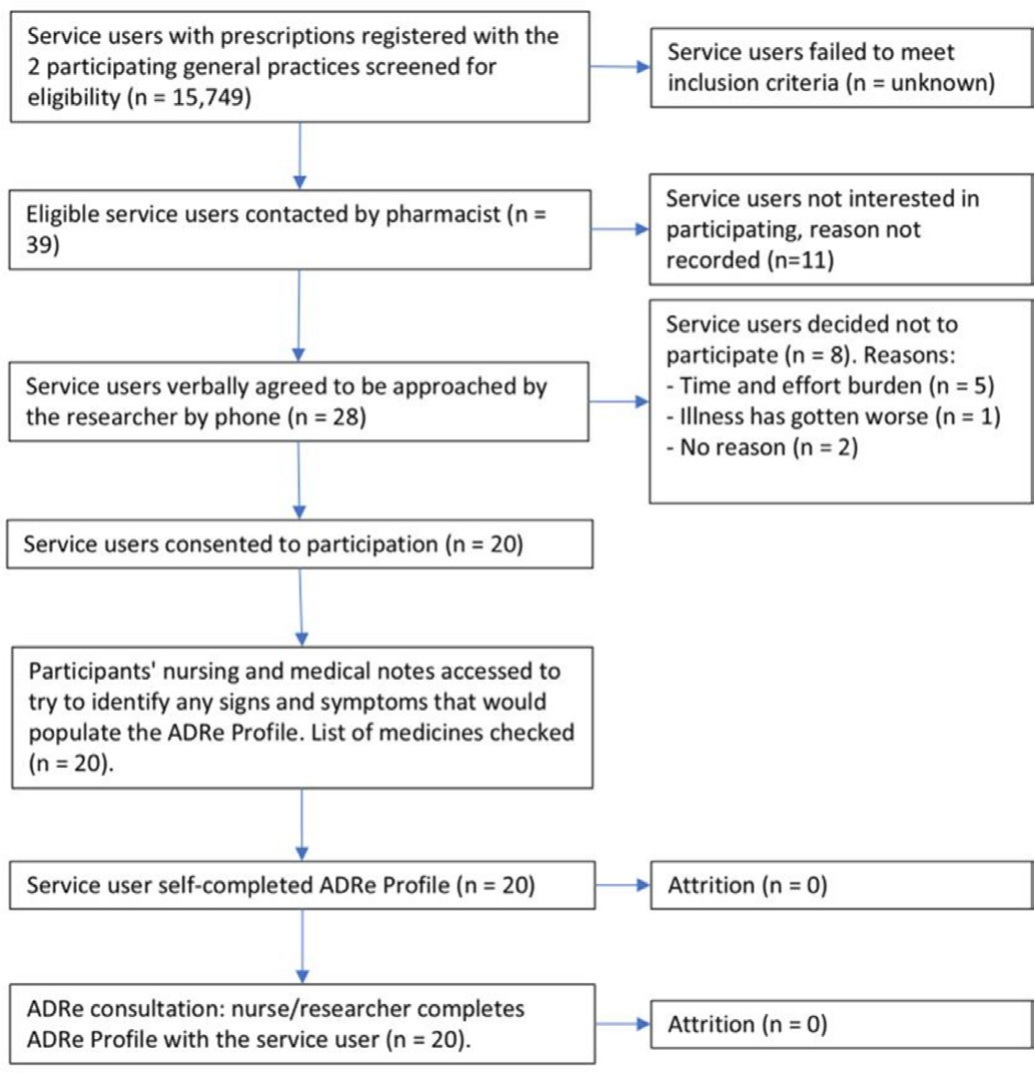
Feasibility test participant flow chart. ADRe, adverse drug reaction.

Two of the 213 GP practices contacted (0.94%) were recruited, one serving a semirural community with 7529 registered patients, and one an urban area with 8153 registered patients.^49^ Of 39 patients approached by the practices, 20 (51.3%) were recruited (10 patients from each) between July 2021 and March 2022 and followed until April 2022. Patients were not asked to give reasons for declining to participate. We had no information on the overall numbers of patients in the practices meeting the inclusion criteria. Figure 2 illustrates participant flow.

Neither practice was in an affluent area. One practice served a former coal-mining community. These less affluent patients reported more problems (table 4).

There was no attrition or loss to follow-up.

**Table 4:** Feasibility test participants’ demographics

	GP practice 1	GP practice 2	Total
WIMD 2019 rank (Welsh Government, 2019)^82^*	734 (2nd most deprived quintile)	972 (3rd, central quintile)*	N/A
Number of participants recruited	10	10	20
Male to female ratio	7:3	2:8	9:11
Age mean (SD)	71.70 (5.85)	75.30 (5.52)	73.50 (5.84)
Number of medicines taken per participant–median (25th–75th centile)	7 (5-9)	8 (5.75–11)	7.5 (5.25–10.5)
Number of problems identified on ADRe–median (25th–75th centile)	11 (8.75–11)	8 (2.75–24)	10 (6–20)

* Ranks and quintiles (WIMD, 2019): 1–382 1st quintile (20% most deprived), 383–764 2nd quintile (20%–40% most deprived), 765–1146 3rd quintile (40%–60% most deprived), 1147–1528 4th quintile (60%–80% most deprived), 1529–1909 5th quintile (20% least deprived).

ADRe, adverse drug reaction; GP, general practice; N/A, not available; WIMD, Welsh Index of Multiple Deprivation.

### Adherence to study procedures

High clinician workload led to the nurse-researcher completing all data collection. Patients’ suboptimal computer/internet literacy and availability (possibly related to costs and broadband coverage) prevented use of the digital ADRe platform prepared by Swansea University. There was full adherence to the study protocol (online supplemental tables 1 and 2). Researchers reviewed completed ADRe Profiles and passed recommendations to patients (health promotion issues only) and clinicians (including concerns regarding some potentially serious signs and symptoms) (online supplemental file 3 has some examples). However, there were no responses to letters addressed to GPs.

### Feasibility of patient self-completion of the ADRe profile

The rate of self-completion of the 79 ADRe items relevant to all patients was 98.29%. 59 ADRe items were completed by all patients (eg, ‘pain’, ‘abnormal posture’, ‘constipation’). 15 ADRe items were not completed by one patient (including ‘skin rash’, ‘chest pain’, ‘confusion’), 3 ADRe items had missing data from 2 patients (including ‘alcohol intake’, ‘reproductive system problems’—these were discretionary/optional items) and 2 ADRe items were not completed by 3 patients (‘high salt intake’ and ‘non-verbal pain signs’). No items were omitted by 10 patients. One participant completed ADRe online.

All 20 participants opted for telephone over videocalls. Items not completed with all participants were: ‘Non-verbal signs of pain’ (17 non-completions, due to inability to see the patient); ‘Reproductive system problems’ (6 non-completions, due to the question being discretionary and researcher’s judgement regarding appropriateness); ‘Abnormal gait’, which was overlooked on one profile; and ‘Immunisations up to date,’ when one patient could not remember whether they had received all recommended immunisations.

### Information overlap between ADRe and patient records

Pre-existing information in the electronic patients’ records held in the GP practices was compared with data collected on the ADRe Profile.

Overall, there was a 22% overlap between the records and the vital signs section of the ADRe Profile. ‘Sitting blood pressure’ and ‘Weight/BMI’ were always recorded in the patient records (100%), ‘orthostatic change in the blood pressure’ was never reported in the patient records (online supplemental table 3), despite 6/20 patients prescribed diuretics and 7/20 prescribed beta blockers (Bisoprolol, Carvedilol); 4/20 were prescribed both a beta blocker and a diuretic. Some records were recent (for two patients sitting BP had been recorded within the past month), but some patients’ BP had been last recorded over 35 months earlier (participants 112 and 211).

Overall, 27 (9.34%) of the 289 ADRe-identified problems (in 20 patients) were also recorded in the patient records. There were seven instances where a problem was recorded in the patient records but not on ADRe. These discrepancies appeared to be attributable to the time difference between the date of the problem and ADRe completion.

### Clinical impact of the ADRe profile

We explored whether the ADRe intervention might alleviate clinical problems.

In all, 289 problems were identified across the ‘Observations’ and ‘Reports’ sections of the ADRe Profile for the 20 participants. The academic research team made 164 recommendations for further investigations or monitoring of the problems and 126 suggestions for actions that would potentially improve or solve the identified problems. The problem most frequently reported was pain (n=16/20), followed by low energy (11/20), and dyspnoea, respiratory or hearing problems (10/20 for each). No-one self-reported: tongue tremor, behaviour problems, high salt intake or constipation. Investigations were recommended for 46 different problems, particularly reports of pain (11 patients), dizziness or ataxia (8 patients), bleeding/bruising or cognitive decline (7 patients), oedema (5 patients). 6/20 participants reported bowel problems, 4/20 reported falls in the past month, 7/20 reported bleeding or bruising and 6/20 reported chest pain.

Suggestions for changes (both pharmacological and non-pharmacological) were made for 43 problems, including 11 for pain (eg, review analgesia), 7 for cognitive decline (eg, check oxygen saturation, review sedative medicines, such as codeine), 6 each for oedema (eg, resting posture) and dizziness (eg, check postural hypotension), 5 for bleeding/bruising (eg, review anticoagulants, check platelets). Some examples are in online supplemental tables 4 and 5. Feedback from participating surgeries was sparse, due to staff sickness (practice 1) and non-response (practice 2). Several participants were referred to specialists for long-term issues. For example, appointments were made for participants 101 and 114 for medication reviews to improve breathlessness (dyspnoea) that was interfering with activities of daily living. Participant 117 used her completed ADRe as a basis for a GP appointment, where bleeding (likely due to high dose of anticoagulant) was resolved, and diuretic dose was rationalised to reduce risk of falls. There was no feedback on our recommendations to the team caring for 202, but there was considerable potential to address the problems of seizures and falls (online supplemental table 4). Review of patient records did not identify where problems were recorded and monitored, other than ADRe. The high prevalence of problems manageable in primary care but with the potential to need secondary care (eg, bleeding, falling, incipient heart or respiratory failure) suggested that introduction of ADRe would have important clinical impact.

Using the decision support incorporated within ADRe, academics highlighted how clinical gains might be made, and problems such as Parkinsonism and falls might be prevented. For example, 3 patients with hand tremor were likely displaying incipient extrapyramidal signs (EPS); therefore, the academics suggested review of antidepressants and/or beta2 agonists as contributing factors. Eight of 20 participants identified balance problems. These patients were prescribed antihypertensives or antidepressants; therefore, academics suggested assessing postural hypotension, ‘falls risks’ and risks of bleeding, alongside medication reviews (table 5). A full summary of problems and suggestions found is included in online supplemental table 5.

**Table 5:** Examples of problems and researchers’ suggestions

ADRe item	
Hand tremor	
Reported by	4 of 20 patients
Suggestions for care, investigations, possible solutions and medicines to be reviewed for 3 patients	(Pt 1) Review the need for mirtazapine in view of emerging EPS EPS are emerging, and the patient receives quite a high single dose of mirtazapine daily. However, mood disorders persist, suggesting a review of antidepressant, alongside other problems, such as uncontrolled pain and insomnia. (Pt 2) Review respiratory medicines and sertraline There appear to be signs of Parkinsonism: it is unlikely that shuffling and stooping are attributable to prescribed medicines (but sertraline may well be contributing, and beta2 agonists are occasionally associated with akathisia). The tremor is at least exacerbated by sertraline, theophylline, formoterol and salbutamol. (Pt 3) Review glucose control. Venlafaxine is at a high dose, and the patient has low mood—consider review. The beta blocker is not controlling the tremor. EPS are apparent and may suggest idiopathic Parkinsonism. However, venlafaxine may be a contributory factor.
Possible complications	Impact on ability to perform activities of daily living, namely difficulty with fine motor skills (eg, handwriting and using tools/utensils), problems with self-care and impaired personal hygiene, reduced independence and increased risk of injury.
Balance	
Reported by	8 of 20 patients
Suggestions for care, investigations, possible solutions and medicines to be reviewed for 8 patients	(Pt 1) Falls risk assessment: falls have already occurred. Postural hypotension measurements. Warfarin increases the risk of bleeding on falling. The high doses of anti-hypertensives and mirtazapine should be reviewed when vital signs have been monitored. (Pt 2) Falls risk assessment. Beta2 agonists are linked with dizziness. Dizziness, particularly on standing. Postural hypotension recordings were not located. Falls may be, in part, attributable to beta2 agonists, theophylline and sertraline. Statins may be contributing to pain and, therefore, falls risk. Corticosteroids increase the risk of fractures and bleeding, which will complicate any falls. (Pt 3) Falls risk assessment. Assess postural hypotension. Review sedatives. Ascertain frequency of PRN codeine use. Risk of falling may be considerable, given that the patient is c/o dizziness and poor balance and is taking several antihypertensives plus an SSRI. (Pt 4) Urgent check of postural BP. Review furosemide dose. Anaemia may be causing loss of balance and tiredness. Codeine, if taken, and solifenacin for bladder control may be causing sedation and falls. (Pt 5) Ascertain alcohol and recreational drug use—interactions possible. Obtain a record of falls. Consider checking carbamazepine concentrations. Review sertraline (high dose). Explore pravastatin and muscle dysfunction. Sertraline may be ineffective, is ‘cautioned’ for patients with epilepsy and, at maximum dose, may be causing problems, including posture and movement disorders, falls, bruising, agitation, asthenia, hallucinations, tinnitus and oedema. (Pt 6) Falls risk assessment—risk of bleeding on falling. Assess postural hypotension. Consider muscle weakness in relation to statin. Verapamil may be responsible for EPS and vertigo, and this might be usefully brought to the attention of the cardiologist. (Pt 7) Falls risk assessment. Assess postural hypotension. Review glucose control and medicines. EPS are apparent, which may suggest idiopathic Parkinsonism. Venlafaxine may be a contributory factor. Falls, ataxia and dizziness are reported. The PPI, antidepressant and beta blocker might be reviewed. (Pt 8) Falls risk assessment. Assess postural hypotension. Explore dizziness in relation to diuretics and ramipril. Review of antihypertensives may alleviate ataxia, tinnitus, paraesthesia and incontinence.
Possible complications	Risk of reduced mobility, social isolation, reduced independence and falls with associated injury and bleeding.

ADRe, adverse drug reaction; BP, blood pressure; EPS, extrapyramidal signs; PPI, proton pump inhibitor; PRN, pro re nata, as needed; SSRI, selective serotonin reuptake inhibitor.

### Safety

No adverse events emanating from use of ADRe or conduct of the research were reported.

### Staff costs of ADRe completion

The research nurse spent some 30 min with each participant, at an estimated cost of £23 per participant. In one practice, the pharmacist spent ~15 min reviewing each ADRe Profile, costing £14 per participant, and 3 of 10 patients were either referred to the relevant physicians or, in one case, referred themselves, at a cost of £32 per consultation. No home visits were undertaken. The cost of each ADRe was therefore ~£37, rising to £69 if additional appointments happened. It became apparent that vital signs are not monitored regularly under normal care for people prescribed five or more medicines. Therefore, an extra 5–10 min of care worker time, at ~£4 should be costed, bringing the cost of each ADRe to £40 or £73. These costs were below the £112 per RCT participant offered by HCRW. Staff costs for the feasibility study fell below the £56 allocated only because the research nurse was funded by the trial.

## Discussion

This feasibility study optimised the design of the RCT, ADRe and fieldwork. The contrast between patients’ and professionals’ enthusiasm for medicines monitoring was striking (>50% vs <1% recruited). The overall suitability and manageability of the individual participant recruitment procedure, the research activities, the potential for clinical gain^9 16 18 20^ and low cost of ADRe administration^16^ were confirmed. However, recruitment and implementation needed to adapt to an unsympathetic environment.

### Recruitment and retention

Staff, not patients, were a barrier to recruitment. Recruiting GP practices for research collaboration proved challenging; clinicians acted as ‘gatekeepers’ to patient involvement, despite potential benefits.^50 51^ Dissonance between patients’ and professionals’ perceptions was apparent, reflecting the reported 72% patient dissatisfaction with the National Health Service (NHS) in Wales.^52^

Clinician involvement in research is affected by an underdeveloped research culture,^53^ perceived relevance of trials^54^ and knowledge of the area investigated.^55^ Workforce shortages, coupled with increasing patient numbers per doctor, decreasing numbers of practices^37^ and the low number of GPs per 1000 population (0.87, compared with Organisation for Economic Co-operation and Development (OECD) average of 1.08^56^) contributed to workload pressures^38 39^ and limited time for activities other than service provision.^57^

### Study procedures

For patients, recruitment was straightforward and timely, barring two instances where delays were caused by staff sickness, not unwillingness to participate. Practitioner-patient rapport promotes recruitment, suggesting that the 1% of practices that did participate enjoyed exceptionally good relationships with their patients.^54 55^

### Completion of the ADRe Profile

Inter-rater reliability between patients and research nurse was high for all items with the overall mean kappa value of 0.71, SD 0.17.^58^ Suboptimal technology availability prevented digital completion of ADRe, incongruent with a reported prevalence of 54% people in the UK aged 65+using internet daily in 2020,^59^ COVID-accelerated digitalisation^60 61^ and the Westminster government’s ‘digital by default’ strategy.^62^,^64^ The costs of internet provision and IT equipment deter many people.

### Overlap with primary care records

Implementation of the ADRe Profile revealed many ongoing health problems that had not been documented. ADRe highlighted problems for clinicians’ attention and presented opportunities to investigate, diagnose and prevent serious issues. Certain problems, often dismissed as natural signs of ageing (for example, hair loss, fatigue and cold extremities),^65 66^ can be related to other signs and symptoms that may form a symptom pattern typical of certain conditions (eg, hypothyroidism, which affects 16% of women over 65^67^), highlighting the importance of thorough evaluation.^20^

### Efficacy signals and potential clinical benefits

The ADRe Profile would lead to clinical benefits were researchers’ suggestions adopted. The ubiquitous, pervasive, insidious, potentially reversible problems reported on ADRe by all patients, as elsewhere,^68 69^ if unaddressed, might contribute to the 10%–16%^5 6 70^ of unplanned hospital admissions due to ADRs, 40%–70% of which are considered preventable.^6 70 71^ They might also contribute to the 18% of preventable deaths attributable to medicines, as reported by coroners.^10^ Many suggestions were for small changes in care—‘Looking for the Little Things’—but these add up to improved care and outcomes.^13^ Suggestions regarding opioids and anticoagulants echo high-profile initiatives.^72^ Following completion of the feasibility study, the RCT^73^ was completed, and clinical benefits ensued.

Criteria have been established for medicines monitoring,^12^ including medication safety action plans involving patients and proactive patient monitoring to prevent administration and monitoring errors. To meet these criteria, services will need to adopt and implement comprehensive patient monitoring instruments for early detection and amelioration of problems. ADRe or a similar system is needed to meet the WHO patient safety goals for polypharmacy^11^ and rationalise UK NHS expenditure.^74^ However, primary care providers were reluctant to engage, despite offers of reimbursement by publicly funded research support schemes.

### Costs

At £40–£73 per administration, ADRe appears cost-effective, with the potential to reduce ADR-related hospital admissions, which currently cost the NHS an estimated £2.2bn annually.^7^ For example, treating a fractured hip following a fall costs £10k–25K,^75^ plus £345–£2349^76^ for each day in hospital. However, our calculation was confined to staff time; no expensive medicines were prescribed. Participants in this study shared demographic characteristics with those most likely to be hospitalised for ADRs.^7075^,^77^

### Strengths and limitations

Only exposition of clinical details can offer insights into the challenges of the unaddressed ADR problem, which has avoidably burdened emergency admission departments for decades,^2 5 6 70 71^ and engendered patient dissatisfaction with the NHS.^52^ No tools predict medicine-related hospital admissions.^68^

Many RCTs are not preceded by feasibility studies.^35^ Systematic review of RCTs identified six instruments for identification and amelioration of ADRs in poly-medicated older people in primary care^15^; none were preceded by a feasibility study. Due to suboptimal clinician involvement, we did not fully replicate how ADRe would be applied in real-world practice. We expected clinicians would check vital signs regularly (eg, annually) for poly-medicated patients and review self-completed ADRe Profiles in conjunction with scheduled medication reviews, but researchers were obliged to substitute.

Recruitment challenges reflected systemic pressures in NHS primary care rather than patient reluctance, contrary to reports.^23^,^25^ Flexible implementation strategies, requiring minimal clinician involvement, were essential to gain the support of overburdened GPs. Our decision to proceed was based on practical considerations, without a ‘red, amber, green’ system.^78^

Staff declined interviews, and these reports of pervasive normalisation of potential harm^79 80^ are based on clinical details.

The low numbers of patients and GP practices preclude generalisation of findings. However, there were sufficient participants to check that 60%–100% of patients would potentially benefit from ADRe.

### Implications

The number of problems identified by ADRe highlights a normalised, unmet need that clinicians felt too busy to address; for example, two participants were experiencing potentially serious bleeding while prescribed anticoagulants.

Data collection procedures, the ADRe Profile and outcome measures were safe, appropriate and feasible. At patient level, the research was welcomed, although with paper-based completion. Options for minimal clinician involvement proved essential to secure participation. Overall, this study suggests that the ADRe Profile would improve patient safety in the target population.

## Supplementary material

10.1136/bmjopen-2025-099627 online supplemental file 1

10.1136/bmjopen-2025-099627 online supplemental file 2

10.1136/bmjopen-2025-099627 online supplemental file 3

## Data Availability

Data are available on reasonable request.
