# Supplementary material for: Breaking barriers: feasibility of a cluster randomised trial evaluating an instrument for identifying and ameliorating adverse drug reactions
Source: BMJ Open. 2026 Jan 27;16(1):e099627. doi: 10.1136/bmjopen-2025-099627 (PMC12853475; doi:10.1136/bmjopen-2025-099627)
Supplement: online supplemental file 3 [file bmjopen-16-1-s003.docx]

# Supplementary File 3

- Supplementary Table 1: Scheduled and completed research activities
- Supplementary Table 2: Scheduled and completed patient contact
- Supplementary Table 3: Vital signs in the medical notes
- Supplementary Table 4: Illustrative case reports of the operation of ADRe
- Supplementary Table 5: ADRe-identified problems and suggestions

## Supplementary Table 1: Scheduled and completed research activities

| RESEARCH tasks (performed once) | Tasks, according to protocol | Comments |
| --- | --- | --- |
| Database search (looking for eligible service users) | GP practice/ pharmacist/ ??clinic / practice manager | Significant delays in finding replacement service users (1 week to 7 months) due to pharmacist workload and sickness in 1 practice. No delays in practice delegating this to the practice manager. |
| Patient eligibility check (for exclusions) | GP practice/ pharmacist/ clinic /researcher | Completed by pharmacist, checked by researcher (at the point of medical notes access). |
| Pharmacist training on ADRe Profile | Pharmacist /researcher | Time taken: 28 mins (pharmacist 1) and 11 mins (pharmacist 2). |
| Practice nurse training on ADRe Profile | Practice nurse / researcher (not done) | The researcher administered the ADRe Profile because participating GP practices did not have capacity to deploy a practice nurse or support worker. |

## Supplementary Table 2: Scheduled and completed patient contact

| PER PARTICIPANT ACTIVITIES | Personnel (according to protocol) | Completion status and comments |
| --- | --- | --- |
| Informed consent | Member of clinical team/researcher | Completed by researcher, participating GP practices did not have capacity to engage a clinical team member. |
| Obtain medical history | Researcher, electronically or in person | Completed in person, remote electronic access option posed unnecessary complications for the GP practice. |
| Obtain a list of prescribed medicines from the service user or the practice | 2 researchers | Completed at the point of accessing medical notes. When medical notes were accessed later than ADRe consultation, checking what was taken from the list was challenging. |
| Instructions/education for service user/care giver | Researcher | Completed, no care givers were engaged. |
| ADRe Profile self-completion | Service user, electronically or by post | Completed, out of 20 service users, only one had sufficient competence and internet access to be able to complete the ADRe Profile electronically.  Number of days between accessing patient notes and ADRe self-completion: mean = 64.35 (SD 55.23), range 192 (min 7, max 199). Reason for long gaps – pharmacist’s sickness and high workload. |
| Administration of the ADRe Profile (ADRe consultation)  In person completion was impossible, due to COVID restrictions. | Practice nurse or researcher using video, or telephone calls, within 2 weeks of service user self-completion | Completed by researcher, participating GP practices did not have capacity to engage a practice nurse. All participants chose to have their ADRe consultation completed by telephone.  Number of days between ADRe self-completion and ADRe consultation: mean = 17.10 (SD 11.09), range 50 (min 6, max 56). There were 2 instances where the gap between data collections was longer than two weeks, the reason was patient unavailability. |
| Pharmacist review of ADRe recommendations and the list of medicines | Pharmacist (academic pharmacist and a professor as a substitute in case of pharmacist unavailability) | Completed by clinical pharmacist in one practice (after receipt of preparatory ‘Review tables,’ proposed by the academic team), but no feedback was provided to researchers. Second practice pharmacist was not able to complete due to excessive workload. Academic pharmacist and a professor reviewed all ADRe Profiles, and provided feedback and recommendations to the clinical teams. |
| GP review (notifying GP of any results/ recommendations) | GP | Researcher sent a concise summary of ADRe findings to GP practices, practice managers have passed the information onto GPs, no feedback was provided from GPs. |
| Participants’ debrief and dissemination of study results | Researcher | Completed, researcher sent a thank you letter and information about the study results to all participants. Due to lack of engagement from the clinicians, clinical debrief about recommended changes or suggestions was not possible. Researcher advice was limited to the “Prevention and health promotion” items. |

## Supplementary Table 3: Vital signs in the medical notes

| ADRe item | Number of participants who had the item recorded in medical notes | Proportion of participants (n=20) with the ADRe item recorded in medical notes |
| --- | --- | --- |
| Vital signs |  | 22% |
| BP sitting | 20 | 100% |
| BP standing | 1 | 5% |
| Pulse | 2 | 10% |
| Weight | 20 | 100% |
| Change in weight | 1 | 5% |
| Bmi | 19 | 95% |
| Height | 1 | 5% |
| O2sat | 1 | 5% |
| ECG | 1 | 5% |
| Irregular rhythm, BP change (sitting and standing), temperature, change in oxygen saturation, lung function tests | 0 | 0 |

## Supplementary Table 4: Illustrative case reports of the operation of ADRe

| Participant ID | Medicines prescribed | Problems identified | Likely causes | Practice changes | ADRe recommendations |
| --- | --- | --- | --- | --- | --- |
| 101  gentleman aged 70-80,  2 problems reported | - **Sacubitril 24mg/ valsartan** 26mg tabs, 1bd - **Rivaroxaban** 20mg 1 daily with food - **Spironolactone** 25mg 1 od - **Zapain®** 30mg/500mg tabs, 1 or 2, 4-6 hourly, PRN, max 8 in a day - **Finasteride** 5 mg tabs, 1 od - **Bisoprolol** 1.25mg tabs, 1 od - **Dapagliflozin** 10mg tabs, 1 od   OTC: none  Prescribed medicines not taken in the last 2 weeks: none. | Bruising  Dyspnoea | Anticoagulant,  Thrombocytopenia possibly due to spironolactone or valsartan  Bisoprolol  Codeine  Heart failure worsening | Appointments made for anticoagulant and COPD reviews | - *Monitor vital signs and symptoms of blood loss, check for thrombocytopenia.* - Bruising in a patient using anticoagulants may indicate a risk of bleeding, which should be investigated. - *Inquire about cough in case linked to dyspnoea.* - Breathlessness on exertion may suggest under-treated heart failure: the continuing need for beta blockers might be considered. |
| 114  gentleman aged 70-80.  Of the 36 problems reported, he prioritised confusion and falls. | - **Simvastatin** 20mg, 1 nocte - **Sertraline**, 1 bd, dose not found in records - **Finasteride** 5mg, 1 od - **Carbocisteine** 375mg capsules, 2 tds - **Prednisolone** 5mg, 6 tabs daily for 5 days for COPD exacerbations - **Uniphyllin** (theophylline) continus® 400mg tabs, 1 nocte - **Ventolin Evohaler** ® (salbutamol)100mcg/dose, inhale 2 doses PRN - **Trimbow®** 5mcg/ 9mcg/ 87mcg/ dose inhaler, 2 puffs BD (formoterol/ glycopyrronium bromide/ beclometasone) - **Paracetamol** 500mg tabs, 1-2 every 4-6hrs PRN - **Cyanocobalamin** 50mcg tabs, 2 od - **Aproderm®** emollient cream PRN - **Folic acid** 5mg tabs, 1 od for 3 months then stop   OTC: **Kalms®** (valerian root extract), **Loratidine** (antihistamine)  Prescribed medicines not taken in the last 2 wks: none. | Confusion, plus dyspnoea with wheezing  Falls, dizziness on standing, muscle weakness  Pain (muscles)  Chest pain | Hypoxia  If prednisolone is used regularly, alongside beclomethasone, it may be contributing to confusion, agitation, mood fluctuations and anxiety.  Other iatrogenic possibilities include sertraline, simvastatin, salbutamol & formoterol causing anxiety, and self-medication with valerian and antihistamines.  Sertraline, corticosteroids, simvastatin, theophylline, beta2 agonists  Simvastatin,  beta2 agonists, theophylline | Referral to geriatrician, who deprescribed theophylline. | - Monitor oxygen saturation & lung function tests. - Consider the management of COPD and the risks of chest infections. - Consider the possibility of incipient heart failure. - Consider the benefit/ harm balance of sertraline. - Record any postural hypotension, which may be due to sertraline or beta2 agonists. - Check a recent ECG for abnormalities associated with antidepressants or beta2 agonists. - Review the use of salbutamol in relation to chest pain. |
| 117  Female, aged 80-90, 9 problems. She prioritised bleeding and the risk of falling. | - **Apixaban**, 2.5mg bd, (lower dose due to bleeding with 5mg – review annually) - **Furosemide** 40mg tablets, 1 od - **Solifenacin** 5mg tablets, 1 od (antispasmodic, anticholinergic) - **Zapain®** 30mg/500mg tablets, 1or 2, 4-6 hourly PRN - **Laxido Orange®** oral powder sachets sugar free - **Adcal D3®**750mg/ 200unit caplets, 1 od   **OTC:** Paracetamol, Rennie  Prescribed medicines not taken in the last 2 weeks: none | Bleeding from (reported) haemorrhoids  Poor balance, and history of falls  Joint pain  Tinnitus  Indigestion | Anticoagulant, without monitoring.  Paracetamol likely above the recommended doses for a small lady  Furosemide, codeine, solifenacin may be causing confusion.  Consider gout from furosemide  Furosemide  Solifenacin, calcium tablets, overuse of antiacids | Participant 117 took the completed ADRe to the doctor at her earliest opportunity. Doses were reduced and problems resolved. | - Check FBC and review anticoagulant. Anaemia may be causing loss of balance and tiredness. - Ensure the recommended dose of paracetamol (OTC + prescription) is not exceeded, particularly if weight <60kgs. - Record postural hypotension. Review furosemide dose. - Codeine may be causing sedation and falls, along with the antimuscarinic for bladder control. - Furosemide may be causing tinnitus, either directly or by dehydration causing earwax to solidify. Please check external auditory meatus. - Furosemide is likely intensifying continence problems. |
| 202  lady aged 60-70  Of 25 problems reported, she prioritised seizures, falls, and depression. | - **Sukkarto®** (metformin) 1g x 2 od - **Alogliptin** 25mg od - **Pravastatin** 20mg 1 nocte - **Sertraline** 100mg x2 od - **Carbamazepine** 200mg x 2 bd = 800mg   OTC: none  Prescribed medicines not taken in the last 2 weeks: none | Abnormal movements at rest  Balance/ coordination  Cognitive decline  Bruising  Seizures (passed out when shopping)  falls  Very low mood | Sertraline and carbamazepine  Sertraline and carbamazepine, under-treated diabetes  Sertraline  Carbamazepine may worsen petit mal and cause syncope.  SSRI overdose may cause seizures and SSRIs are contra-indicated in poorly controlled epilepsy.  Low mood may be due to carbamazepine, pravastatin or sertraline or any recreational drug use. | None reported. | - Sertraline at this maximum dose may be contributing to seizures, movement disorders, falls, bruising, agitation, asthenia, hallucinations, tinnitus, oedema. - Check carbamazepine concentrations. - Arrange seizure and falls diaries. Relate to capillary glucose.      - There were no responses to alcohol and recreational drug use questions, and these warrant follow up. |

Abbreviations: od - once daily, bd - twice daily, PRN - as needed, OTC - over the counter medicines

Practice 1 closed March 2024, following GP resignations September 2023.

## Supplementary Table 5: ADRe-identified problems and suggestions

| ADRe item (sample) | Number of participants who identified ADRe item as present | Number of times further investigations were suggested | Number of times solutions were suggested | Example of the academic team’s suggestions as to medicines to be reviewed |
| --- | --- | --- | --- | --- |
| Hand tremor (n=20) | 4 | 2 | 3 | Review the need for mirtazapine in the light of emerging extra-pyramidal symptoms. |
| Feet shuffling (n=19) | 3 | 0 | 2 | Movement disorders a concern with venlafaxine pregabalin. |
| Abnormal movements at rest (n=20) | 4 | 0 | 4 | Please review sertraline –  maximum dose prescribed. Sertraline may also be related to the neuromuscular dysfunction described and the referral arranged. Despite max dose, the patient has low mood. |
| Posture abnormal (n=20) | 3 | 0 | 1 | Movement disorders a concern with venlafaxine |
| Gait abnormal (n=18) | 5 | 0 | 3 | Consider when reviewing cardiac medicines. Feet shuffling reported, making EPS more likely – urgent review of verapamil. ? others |
| Balance problems (n=20) | 8 | 8 | 5 | Beta2 agonists are linked with dizziness, below. |
| Cognitive decline (n=20) | 7 | 6 | 7 | anti-depressant and statin. |
| Feeling cold (n=20) | 6 | 3 | 2 | Morphine may be suppressing the regulatory centres, contributing to hypothermia. |
| Bleeding, bruising (n=19) | 7 | 6 | 5 | Co-dydramol should be considered. Inhaled corticosteroid possibly contributory. |
| Skin rash (n=19) | 5 | 2 | 3 | Review rabeprazole, particularly if >8 weeks. Almost any medicine can cause a rash. |
| Swelling, oedema (n=20) | 6 | 5 | 6 | Sodium content of medicines and diet should be reviewed. Allopurinol, montelukast a remote possibility. |
| Sweating (n=20) | 4 | 4 | 2 | Consider hypoglycaemia due to ??. |
| Acne (n=19) | 3 | 2 | 0 | Not represented. |
| Broken skin, poor healing (n=20) | 5 | 3 | 1 | Consider immunosuppression by opioids - remote. |
| Hair loss (n=20) | 9 | 3 | 2 | Benefits may outweigh the disadvantages of allopurinol & statins. |
| Skin abnormalities (n=20) | 4 | 0 | 0 | Review anticoagulant and Statin. Consider prescribing emollients. |
| Injection site problems (n=20) | 2 | 0 | 0 | Not represented. |
| Convulsions (n=20) | 1 | 1 | 1 | Review epilepsy management. There is a note saying ‘petit mal’ – carbamazepine does not treat petit mal. Carbamazepine can cause syncope. Consider SSRI withdrawal. SSRI overdose (this dose is very high) can cause seizures and SSRIs are contra-indicated in poorly controlled epilepsy. |
| Behavioural problems (n=19) | 0 | 0 | 0 | Not represented. |
| Self-harm (n=20) | 0 | 0 | 0 | Not represented. |
| Physical violence (n=20) | 1 | 1 | 1 | Consider beta2 agonists / sympathomimetic. |
| Aggression (n=20) | 3 | 1 | 1 | Review antidepressant. |
| Agitation, anxiety (n=20) | 6 | 6 | 3 | Consider ramipril & zopiclone. Could this anxiety be agitation caused by lamotrigine? |
| Restlessness (n=20) | 5 | 2 | 3 | Review sertraline. |
| Panic attacks (n=20) | 6 | 1 | 0 | Not represented. |
| Confusion (n=19 | 4 | 2 | 2 | Full respiratory / medication review. |
| Mood fluctuations (n=19) | 6 | 3 | 4 | Review carbamazepine, sertraline and statin. |
| Low energy, fatigue (n=18) | 11 | 3 | 4 | Consider codeine. |
| Hallucinations (n=20) | 6 | 5 | 2 | Review antidepressant. |
| Sleep problems (n=19) | 6 | 2 | 1 | Review possible nocturnal hypoglycaemia (also night sweats). Consider administering SNRI in the morning. |
| Excessive sedation (n=20) | 1 | 1 | 0 | Not represented. |
| Dizziness (n=20) | 9 | 8 | 6 | Review combination of antihypertensives. |
| Falls (n=19) | 4 | 4 | 4 | Review antihypertensives. Consider muscle weakness and statins and steroids. |
| Headache (n=20) | 5 | 1 | 0 | Not represented. |
| Pain (n=20) | 16 | 11 | 11 | Review analgesia. Is paracetamol being used? Review application of capsaicin in relation to burning pain. Rivaroxaban is a possible cause of pain in extremities. |
| Non-verbal signs of pain (n=16) | 2 | 0 | 0 | Not represented. |
| Tingling, pins and needles in extremities (n=20) | 8 | 4 | 4 | Consider doses of antihypertensives (all high). |
| Hearing problems, tinnitus (n=20) | 10 | 5 | 3 | Review diuretic, |
| Vision, eyesight problems (n=20) | 9 | 8 | 3 | Review allopurinol. |
| Dry eyes (n=20) | 4 | 2 | 0 | Not represented. |
| Urination problems (n=19) | 6 | 4 | 2 | Review relevant medicines. Consider mid-day administration of furosemide. |
| Catheter problems (n=20) | 0 | 0 | 0 | Not represented. |
| Reproductive system disfunction (n=18) | 5 | 1 | 0 | Not represented. |
| Chest pain (n=19) | 6 | 7 | 1 | Beta2 agonists increase cardiac demands. |
| Short of breath (n=20) | 10 | 7 | 4 | Steroids are likely increasing chest infections. |
| High salt intake (n=17) | 0 | 0 | 0 | Not represented. |
| Dental problems (n=19) | 5 | 3 | 3 | Zopiclone may be causing xerostomia, causing the gums to shrink. |
| Dry mouth (n=20) | 8 | 7 | 2 | Consider artificial saliva preparations |
| Halitosis (n=19) | 3 | 2 | 2 | Diabetes + GERD/ GORD control should be reviewed, as possible causes. |
| Hyper-salivation (n=20) | 3 | 1 | 0 | Not represented. |
| Swallowing difficulties (n=20) | 2 | 3 | 1 | Consider extra-pyramidal symptoms caused by ?? . |
| Indigestion, heartburn (n=20) | 9 | 6 | 5 | Antacids may be causing indigestion. |
| Nausea, vomiting (n=20) | 3 | 1 | 1 | If nausea is a long-standing problem, medication regimen needs a thorough review. |
| Appetite or taste changes (n=20) | 2 | 1 | 1 | Review diet and consider weight loss. Complete diet diary. |
| Bowel control, diarrhoea (n=19) | 6 | 3 | 4 | Consider the value of PPI, and whether iron is still needed. |
| Constipation (n=20) | 0 | 0 | 0 | Not represented. |
| Sore throat (n=20) | 3 | 0 | 0 | Not represented. |
| Respiratory problems developing (n=20) | 10 | 3 | 1 | Beta blocker may be worsening COPD. Consider PPI as a possible cause of the cough, particularly if prescribed >8 weeks. |
