# Supplementary material for: Breaking barriers: feasibility of a cluster randomised trial evaluating an instrument for identifying and ameliorating adverse drug reactions
Source: BMJ Open. 2026 Jan 27;16(1):e099627. doi: 10.1136/bmjopen-2025-099627 (PMC12853475; doi:10.1136/bmjopen-2025-099627)
Supplement: Supplementary file 4 [file bmjopen-2025-099627.reviewer_comments.pdf]

PEER REVIEW HISTORY

BMJ Open publishes all reviews undertaken for accepted manuscripts. Reviewers are asked to complete a checklist review form and are provided with free text boxes to elaborate on their assessment. These free text comments are reproduced below.

ARTICLE DETAILS

Title (Provisional)

Breaking barriers: Feasibility of a cluster randomised trial evaluating an instrument for identifying and ameliorating adverse drug reactions

Authors

Logan, Vera; Hughes, David; Turner, Adam; Carter, Neil; Jordan, Sue

---

VERSION 1 - REVIEW

Reviewer **1**

Name **Liang, Xiaohua**

Affiliation **Chongqing Medical University Affiliated Children's Hospital Department of Respiratory, Clinical Epidemiology and Biostatistics Department**

Date **07-Mar-2025**

COI **None**

First, the statistical method of this study was so sample, the author may compare the response between sex, age group or other characteristics.

Second, images could be used for statistical description.

Reviewer **2**

Name **Bennett, Kathleen**

Affiliation **Royal College of Surgeons in Ireland, Population Health Sciences**

Date **29-Jul-2025**

COI **None**

The paper is based on a feasibility assessment of an RCT evaluating an instrument for identifying adverse drug reactions and improving GP engagement. The study uses the ADRe profile in general practice for older adults prescribed multiple medicines. It is a small study of only 20 participants, therefore, challenging in drawing too many conclusions from this.

There are some comments for consideration.

Two GP practices (0.94% of 213 approached) were recruited which is a very small number. What was the original number of practices expected to be recruited? Also 20 participants from the two practices appears a very low number even for a feasibility study.

In the introduction the authors refer to costs of ADR 'extrapolated over 12 months, this implies a cost of £2.21bn from a total Department of..' However, it was not clear how was this extrapolated and for what year?

The aim of the study 'to evaluate the feasibility and acceptability of using the ADRe Profile in general practice surgeries for older adults prescribed multiple medicines.' However, PICO was not applied here; was there a comparator group ?

The last sentence of the introduction would be better placed in the discussion, not the introduction.

In the methods, the authors state that there were 114 invitations but there were 226 practices. It is not clear whether all practices were invited or a subset and the abstract refers to 213 approached. The numbers should be consistent throughout.

The study recruitment was 1st July 2021 and 15th March 2022 which is almost 4 years ago. Is there a reason for the delay in reporting this study?

For outcomes in table 2 what is the numerator and denominator information? Analysis should include 95% Confidence intervals. Also, there is no mention of qualitative data and how this will be analysed.

In the results, it refers to 39 patients approached by the practices, and 20 recruited but how were the 20 chosen from the 39? Randomly selected or other?

The number of medicines taken per participant and Number of problems identified on ADRe – it might be better to present as median (IQR) rather than means as the data are likely to be skewed data (lower threshold effect), and mean number of medicines/problems results in non-integer values.

The initial aim 'to evaluate the feasibility and acceptability of using the ADRe Profile in general practice surgeries' however the results include the clinical impact of ADRe profile (which was not included in original aims) and also the costs (also not in the aim).

In the discussion, there is limited reference to other similar studies for comparison – and how these compared, strengths and weaknesses, to the current study.

There were no strengths mentioned and the small size of the study(20 participants) may have limited the findings and conclusions from the study.

Implications could be explored further in relation to whether a definitive trial is required, or further research in this area.

**Reviewer**	**3**
**Name**	**Wright, David**
**Affiliation**	**University of Leicester, School of Healthcare**
**Date**	**09-Sep-2025**
**COI**	**None**

This paper describes the results from a feasibility study to assess whether a tool for identifying medication related problems and preventing adverse drug reactions is feasible.

The study consists of two medical practices and 20 patients and even as a feasibility study is small. Recruitment of medical practices was very low and therefore it could be argued that an RCT, if clustered at practice level, would not be feasible. Due to concerns regarding washover of such an intervention on practitioner behaviour of using such a tool would mean that randomisation would have to be at healthcare professional level as the bare minimum. Consequently, any reasonably powered RCT would require participation by a reasonable number of medical practices.

Whilst there is no argument that ADRs are a major cost for the health system, a good proportion of these are not preventable. Medicines are prescribed knowing that there are risks associated with them which cannot be predicted or prevented, consequently such research is probably more interested in preventing preventable adverse drug events. The introduction, as currently written, needs to be more accurate in its description as to what can be prevented by such tools.

The authors report that the tool has been shown to be effective in previously reported RCTs however both studies report on process measures which presuppose patient benefits but not on any clinical outcome measures which would usually be associated with the concept of effectiveness. The authors therefore need to clarify that the tool has been shown to be effective at identifying medication related problems which in theory should reduce future adverse drug events.

As these were RCTs the next question is why a feasibility study is required and therefore a better argument for this study is perhaps required.

A rationale that 20 patients would be enough for a feasibility study is insufficient. A rationale based on accuracy of final estimates and ability to effectively assess progression criteria is now usual practice and would be expected here. I suspect 20 is not enough.

Was the protocol registered with a trials database prior to study completion? It would be useful to be able to compare initial intentions with final feasibility outcomes.

The use of the term ‘outcomes’ for objectives is unusual in such a protocol. The outcomes listed here, would usually form the basis for progression criteria.

Some of the objectives would form the basis of assessing intervention dose, reach and fidelity. The authors do not seem to be using language now usually seen in clinical trials of this nature.

I would expect a range of outcome measures to be tested for feasibility of collection and this to be informed by the COMET database.

The authors plan to inform the delivery of an RCT which has process outcome measures, which in reality no funder would support. Process measures are used to explain the changes in measured clinical outcomes and not as primary outcome measures. An important element of a feasibility study is the ability to capture clinical outcomes i.e. patient willingness to complete them or be assessed clinically.

Progress criteria are usually presented as red, amber, green. The criteria presented here are not presented as anticipated and seem to be selected to enable feasibility to be demonstrated. Within the methods 60% of patient agreement to participated was anticipated but in the progression criteria 40% was set as acceptable.

The approach to costing is simplistic and should be informed by a health economist. As presented this data is of very limited use and probably should not be reported beyond the ability to estimate staff time contributions. Cost and resource use would also include medication costs, hospitalisation costs, GP visit or time costs. None of these are included in the analysis.

Demographic data such as number of medicines and problems is usually skewed and as such median (IQ) better represents the data.

This study is four years old and is heavily reliant on the use of paper and therefore may be of less relevance in a world where everything is not computer based and generated. In fact the question today would be whether AI could use this tool to generate the problem list.

The results assess patient ability to complete the form themselves but I could not see this objective or progression criteria in the methods.

Only one interview was undertaken which is insufficient to understand the acceptability and practicality of using the tool within a feasibility study of this nature.

The ability of the tool to identify clinical problems has already been demonstrated and I am unsure what the value of the academic research team review of the data adds to the literature or this as a feasibility study.

There is an over emphasis on the possible impact of the tool, which I would not expect to see in so much detail. The authors seem overly focused on showing the value of their approach rather on the feasibility of a future RCT.

I don't believe that, as presented and designed, this study demonstrates the need for the study, provides evidence that the intervention is acceptable or practical, that a trial could be

completed due to low recruitment rate and that it could be used to inform a future trial, due to the lack of clinical outcome measures.

VERSION 1 - AUTHOR RESPONSE

	Reviewer: 1	
	Dr. Xiaohua Liang, Chongqing Medical University Affiliated Children's Hospital Department of Respiratory	
	Comments to the Author:	
1.	First, the statistical method of this study was so simple, the author may compare the response between sex, age group or other characteristics.	Only descriptive statistics were possible. We did not aim to analyse the demographic or other differences between the 2 practices, and do not understand the purpose in such analyses. There are no inferential analyses in this paper.
2.	Second, images could be used for statistical description.	There are no "statistical descriptions". We do not know what is meant by "images for statistical description", and the editor did not explain.
		We are pleased that reviewer 1 had no other negative comments.
	Reviewer: 2	
	Prof. Kathleen Bennett, Royal College of Surgeons in Ireland	
	Comments to the Author:	
3.	The paper is based on a feasibility assessment of an RCT evaluating an instrument for identifying adverse drug reactions and improving GP engagement. The study uses the ADRe profile in general practice for older adults prescribed multiple medicines. It is a small study of only 20 participants, therefore, challenging in drawing too many conclusions from this.	We agree. We conclude only that ADRe is feasible and holds promise in this population.
	There are some comments for consideration.	
4.	Two GP practices (0.94% of 213 approached) were recruited which is a very small number. What was the original number of practices expected to be recruited? Also 20	Thank you. It should be borne in mind that the feasibility study was a small preliminary component of the larger RCT. We felt it was worth describing this aspect of the study in detail as

	participants from the two practices appears a very low number even for a feasibility study.	published accounts of the conduct of feasibility studies are largely absent from the literature. We should have clarified that small non-randomised feasibility studies typically do not have formal sample size calculations (Eldridge et al 2016). We have added under 'limitations':<br>The low numbers of patients and GP practices in this study precludes generalisation of findings to other settings. However, there were sufficient participants to check that 60%-100% of patients would potentially benefit from ADRe; only 15 participants were needed to ascertain a prevalence within 95% confidence intervals of 60% to 100% (Uitenbroek 1997).
5.	In the introduction the authors refer to costs of ADR 'extrapolated over 12 months, this implies a cost of £2.21bn from a total Department of..' However, it was not clear how was this extrapolated and for what year?	We apologise for this lack of clarity. Osanlou et al 2022 extrapolated data for non-elective admissions in NHS England 2018-2019.
6.	The aim of the study 'to evaluate the feasibility and acceptability of using the ADRe Profile in general practice surgeries for older adults prescribed multiple medicines.' However, PICO was not applied here; was there a comparator group ?	Thank you for identifying this ambiguity. There was no comparator group. We have now clarified: A single-arm feasibility study …
7.	The last sentence of the introduction would be better placed in the discussion, not the introduction.	Thank you for the attention to detail. This sentence has been moved.
8.	In the methods, the authors state that there were 114 invitations but there were 226 practices. It is not clear whether all practices were invited or a subset and the abstract refers to 213 approached. The numbers should be consistent throughout.	We apologise. We should have been clearer. We approached both 226 practices and 114 individuals. 13 practices were non-contactable. Informally, we attributed this to the high rate of closures of practice in Wales since 2012.
9.	The study recruitment was 1st July 2021 and 15th March 2022 which is almost 4 years ago. Is there a reason for the delay in reporting this study?	The RCT was part of a PhD project, with time-limited funding. Under our regulations, the PhD must be successfully examined before data

		can be place in the public domain. The submission to BMJ open was shortly after the thesis was accepted in 2024.
10.	For outcomes in table 2 what is the numerator and denominator information? Analysis should include 95% Confidence intervals. Also, there is no mention of qualitative data and how this will be analysed.	Thank you, we have clarified.<br>Table 2 is not a numerical table. We did not set strict criteria, and now state: no proportions were pre-specified.<br>No qualitative data were presented and analysed in this paper. We have removed reference to the interview.
11.	In the results, it refers to 39 patients approached by the practices, and 20 recruited but how were the 20 chosen from the 39? Randomly selected or other?	Thank you. We have now stated: Patients were not asked to give reasons for declining to participate.
12.	The number of medicines taken per participant and Number of problems identified on ADRe – it might be better to present as median (IQR) rather than means as the data are likely to be skewed data (lower threshold effect), and mean number of medicines/problems results in non-integer values.	Thank you. We thought it best to be consistent and report the same measure of central tendency across the variables. We agree that some parameters are not normally distributed.<br>We have now reported medians and quartiles.
13.	The initial aim 'to evaluate the feasibility and acceptability of using the ADRe Profile in general practice surgeries' however the results include the clinical impact of ADRe profile (which was not included in original aims) and also the costs (also not in the aim).	These are aims 5 and 6.
14.	In the discussion, there is limited reference to other similar studies for comparison – and how these compared, strengths and weaknesses, to the current study.	Since submission, our systematic review has been published. We are now able to add: Systematic review of RCTs identified 6 instruments for identification and amelioration of adverse drug reactions in polymedicated older people in primary care (Logan et al 2025); none of these RCTs was preceded by a feasibility study.<br>Consequently, our discussion has focussed largely on the methodological literature.

15.	There were no strengths mentioned and the small size of the study(20 participants) may have limited the findings and conclusions from the study.	We agree, and have added the low numbers to the 'limitations' section.
16.	Implications could be explored further in relation to whether a definitive trial is required, or further research in this area.	Thank you. We have now referred readers to other papers where implications are explicated. We were conscious of the need to comply with word limits.
	Reviewer: 3	
	Dr. David Wright, University of Leicester	
	Comments to the Author:	
17.	This paper describes the results from a feasibility study to assess whether a tool for identifying medication related problems and preventing adverse drug reactions is feasible.	Thank you.
18.	The study consists of two medical practices and 20 patients and even as a feasibility study is small. Recruitment of medical practices was very low and therefore it could be argued that an RCT, if clustered at practice level, would not be feasible. Due to concerns regarding washover of such an intervention on practitioner behaviour of using such a tool would mean that randomisation would have to be at healthcare professional level as the bare minimum. Consequently, any reasonably powered RCT would require participation by a reasonable number of medical practices.	Thank you for this advice. We are unsure how it relates to the paper in hand. The RCT to which the feasibility study relates has been completed.<br>We note the size of the study and resource constraints under limitations.
19.	Whilst there is no argument that ADRs are a major cost for the health system, a good proportion of these are not preventable. Medicines are prescribed knowing that there are risks associated with them which cannot be predicted or prevented, consequently such research is probably more interested in preventing preventable adverse drug events. The introduction, as currently	This is an important point, thank you. We have augmented the introduction accordingly.

	written, needs to be more accurate in its description as to what can be prevented by such tools.	
20.	The authors report that the tool has been shown to be effective in previously reported RCTs however both studies report on process measures which presuppose patient benefits but not on any clinical outcome measures which would usually be associated with the concept of effectiveness. The authors therefore need to clarify that the tool has been shown to be effective at identifying medication related problems which in theory should reduce future adverse drug events.	We are unsure as to the relevance of this comment to the paper submitted. In both RCTs 'actions taken' was an important outcome. In the RCT in care homes ADRe reduced the number of problems patients experienced, including pain, falls and infections. These are clinical outcomes, not process outcomes, and are important to patients.
21.	As these were RCTs the next question is why a feasibility study is required and therefore a better argument for this study is perhaps required.	Thank you, we have stated the clinical areas of the earlier research and clarified:<br>"feasibility studies in the target clinical area are recommended as a precursor to full-scale trials".<br>This study is in a different clinical area to the previous work, and feasibility would not be transferable.
22.	A rationale that 20 patients would be enough for a feasibility study is insufficient. A rationale based on accuracy of final estimates and ability to effectively assess progression criteria is now usual practice and would be expected here. I suspect 20 is not enough.	We agree that we should have addressed this concern. We have now written:<br>A sample size calculation was not undertaken for this non-randomised feasibility study, as elsewhere (Eldridge et al 2016).<br>The decision to recruit 20 participants from 2 GP practices was made to provide some variability between urban and semi-rural communities, yet remain practicable within the time and resource constraints of the project.<br><br>We have addressed this point under 'limitations' and added:<br>The low numbers of patients and GP practices in this study precludes generalisation of findings to other settings. However, there were

		sufficient participants to check that 60%-100% of patients would potentially benefit from ADRe; 15 participants were needed to ascertain a prevalence within 95% confidence intervals of 60% to 100% (Uitenbroek 1997).
23.	Was the protocol registered with a trials database prior to study completion? It would be useful to be able to compare initial intentions with final feasibility outcomes.	It was. The registration and citation for the published protocol were given in the paper.
24.	The use of the term 'outcomes' for objectives is unusual in such a protocol. The outcomes listed here, would usually form the basis for progression criteria.	We are uncertain as to the provenance of this comment, but have removed the heading 'outcomes' from Table 2.
25.	Some of the objectives would form the basis of assessing intervention dose, reach and fidelity. The authors do not seem to be using language now usually seen in clinical trials of this nature.	Thank you. We are unable to identify any linguistic or grammatical errors in the manuscript. We have introduced the term 'fidelity' alongside 'adherence'. Our study was too small to speculate on geographical and socio-economic reach. No doses were involved. We can address any specific points of grammar.
26.	I would expect a range of outcome measures to be tested for feasibility of collection and this to be informed by the COMET database.	Thank you. We list the measures to be tested in Table 2. We opted to use EQUATOR guidelines, but would be happy to take advice and add the statement recommended by Kirkham et al 2016. We stated:<br>Reporting was guided by the CONSORT extension to randomised pilot and feasibility trials [25, Supplementary File 1], Guidelines for reporting non-randomised pilot and feasibility studies [38], and the Tidier checklist [39].<br>ADRe does not address any single disease entity, so does not fit easily into the COMET database https://www.comet-initiative.org/ .
27.	The authors plan to inform the delivery of an RCT which has process	The RCT has been funded, and completed.

	outcome measures, which in reality no funder would support. Process measures are used to explain the changes in measured clinical outcomes and not as primary outcome measures. An important element of a feasibility study is the ability to capture clinical outcomes i.e. patient willingness to complete them or be assessed clinically.	The process of care is very important in nursing.<br>We report on patient participation. Retention was 100%.<br>As stated in Table 2, this feasibility study assessed the potential to improve clinical outcomes.
28.	Progress criteria are usually presented as red, amber, green. The criteria presented here are not presented as anticipated and seem to be selected to enable feasibility to be demonstrated. Within the methods 60% of patient agreement to participated was anticipated but in the progression criteria 40% was set as acceptable.	We agree that this is a limitation of the study.<br>Like most (87%) trialists, our decision to proceed was based on practical considerations, without a 'red, amber, green' system (Mellor et al 2021). We have added this statement to the limitations of our study.<br>We agree there is a discrepancy between expected and accepted proportions, as stated but are unsure as to why this is a problem.
29.	The approach to costing is simplistic and should be informed by a health economist. As presented this data is of very limited use and probably should not be reported beyond the ability to estimate staff time contributions. Cost and resource use would also include medication costs, hospitalisation costs, GP visit or time costs. None of these are included in the analysis.	Thank you: we should have clarified, and have now added to the objectives in Table 2:<br>To ascertain whether the potential staff costs of implementing ADRe would be within the research support offered by Health and Care Wales: £56 per participant for the feasibility study and £112 for the RCT.<br><br>GP consultation costs were included. GP visits are almost unknown in our area. The costs of the medicines administered were minimal. We have clarified that no home visits were undertaken, and added to our brief discussion of the costs:<br>"our calculation was confined to staff time; no expensive medicines were prescribed."
30.	Demographic data such as number of medicines and problems is usually	We agree.<br>We thought it best to be consistent and report the same measure of

	skewed and as such median (IQ) better represents the data.	central tendency across the variables. We agree that some parameters are not normally distributed. We now report medians with 25th-75th centiles.
31.	This study is four years old and is heavily reliant on the use of paper and therefore may be of less relevance in a world where everything is not computer based and generated. In fact the question today would be whether AI could use this tool to generate the problem list.	Thank you, it is a good idea to mention this.<br>The RCT was part of a PhD project, with time-limited funding. Under our rules, the PhD must be successfully examined before data can be place in the public domain. The submission to BMJ open was shortly after the thesis was accepted.<br>We have added:<br>"The ADRe tool is now available electronically, and generates a 'problem list' when prompted. "<br>However, older people in our communities continue to prefer paper instruments; many are unable to afford internet connections.
32.	The results assess patient ability to complete the form themselves but I could not see this objective or progression criteria in the methods.	This is objective 3:<br>Items completed / not completed independently by the patient. (Table 2)
33.	Only one interview was undertaken which is insufficient to understand the acceptability and practicality of using the tool within a feasibility study of this nature.	We agree, thank you. We have removed reference to this interview. We shall report the interview elsewhere.
34.	The ability of the tool to identify clinical problems has already been demonstrated and I am unsure what the value of the academic research team review of the data adds to the literature or this as a feasibility study.	Thank you. Use of the ADRe tool has not been reported in GP practices. This setting is very different to the earlier trials.<br>This is objective 5: potential to improve clinical outcomes.
35.	There is an over emphasis on the possible impact of the tool, which I would not expect to see in so much detail. The authors seem overly focused on showing the value of their approach rather on the feasibility of a future RCT.	This is an important point, which we have now raised in the discussion: Many suggestions were for small changes in care, which we describe as 'Looking for the Little Things' (Gwnewch y pethau bachain), but these add up to improved care and outcomes (Hughes et al 2020).

		We feel that it is important to illustrate the possible impact of ADRe. This cannot be done without attention to clinical detail or "looking at the little things" / gwnewch y pethau bachain. These findings refined our clinical outcome measure for the RCT.
36.	I don't believe that, as presented and designed, this study demonstrates the need for the study, provides evidence that the intervention is acceptable or practical, that a trial could be completed due to low recruitment rate and that it could be used to inform a future trial, due to the lack of clinical outcome measures.	The trial was informed by the feasibility study, and has been completed. Patient recruitment rate was not low.<br>We agree that clinicians' views of acceptability and practicability are important barriers to uptake of ADRe, and state this.<br>We believe that our report of the contrast between clinicians and patients is an important contribution to the literature, and will inform readers and the research community.

Eldridge SM, Lancaster GA, Campbell MJ, Thabane L, Hopewell S, Coleman CL, et al. Defining feasibility and pilot studies in preparation for randomised controlled trials: Development of a conceptual framework. PloS one. 2016;11(3):e0150205–e0150205.

Hughes, D.; Jordan, M.; Logan, P.A.; Willson, A.; Snelgrove, S.; Storey, M.; Vaismoradi, M.; Jordan, S. Looking for the "Little Things": A Multi-Disciplinary Approach to Medicines Monitoring for Older People Using the ADRe Resource. *Geriatrics* **2020**, *5*, 79. https://www.mdpi.com/2308-3417/5/4/79

Kirkham JJ, Gorst S, Altman DG, Blazeby JM, Clarke M, Devane D, et al. (2016) Core Outcome Set–STAndards for Reporting: The COS-STAR Statement. PLoS Med 13(10): e1002148. https://doi.org/10.1371/journal.pmed.1002148

Logan V, Hughes D, Turner A, Carter N, Jordan S (2025) Methods for identifying adverse drug reactions in primary care: A systematic review. PLoS ONE 20(2): e0317660. https://doi.org/10.1371/journal.pone.0317660

Mellor K, Eddy S, Peckham N, Bond CM, Campbell MJ, Lancaster GA, Thabane L, Eldridge SM, Dutton SJ, Hopewell S. Progression from external pilot to definitive randomised controlled trial: a methodological review of progression criteria reporting. BMJ Open. 2021 Jun 28;11(6):e048178. doi: 10.1136/bmjopen-2020-048178. PMID: 34183348; PMCID: PMC8240572.

Taylor B, Lobont C, Dayan M, Merry L, Jefferies D, Wellings D. Public satisfaction with the NHS and social care in 2024 (BSA). [Internet]. The King's Fund. 2025. Available from: https://www.kingsfund.org.uk/insight-and-analysis/reports/public-satisfaction-nhs-social-care-in-2024-bsa

VERSION 2 - REVIEW

**Reviewer**	**2**
**Name**	**Bennett, Kathleen**
**Affiliation**	**Royal College of Surgeons in Ireland, Population Health Sciences**
**Date**	**17-Dec-2025**
**COI**	

The authors have responded to the previous comments made. There a couple of addition items to mention.

1. The paper would benefit from a careful proofread for typos. I noted a couple appeared after the revision (e.g. line 234 'illustrates' and 296 should be 'using SPSS')

2. Sample size is referred to in the discussion (under limitations) for the first time. This should be moved to the methods, although the authors state that 'A sample size calculation was not undertaken as this was a non-randomised feasibility study', so this may appear contradictory. I agree one of the other reviewers that sample size is now calculated for feasibility studies (randomised or not) based on progression criteria (see Lewis et al. Pilot and Feasibility Studies (2021) https://doi.org/10.1186/s40814-021-00770-x).

3. line 33 in the discussion - the authors refer to 'Like most trialists..' - I don't think it is appropriate to generalise in this way so would omit this.

VERSION 2 - AUTHOR RESPONSE

Reviewer: 2	
Prof. Kathleen Bennett, Royal College of Surgeons in Ireland	
Comments to the Author:	
The authors have responded to the previous comments made. There a couple of addition items to mention.	
1. The paper would benefit from a careful proofread for typos. I noted a couple appeared after the revision (e.g. line 234 'illustrates' and 296 should be 'using SPSS')	Thank you. We apologise and have corrected typo's and inconsistencies.<br><br>We have removed the preposition and substituted a gerund, as requested. The sentence now reads: undertaken using the Statistical Package for the Social Sciences (SPSS, version 30).

	We have rechecked. Word does not identify any errors.
2. Sample size is referred to in the discussion (under limitations) for the first time. This should be moved to the methods, although the authors state that 'A sample size calculation was not undertaken as this was a non-randomised feasibility study', so this may appear contradictory. I agree one of the other reviewers that sample size is now calculated for feasibility studies (randomised or not) based on progression criteria (see Lewis et al. Pilot and Feasibility Studies (2021) https://doi.org/10.1186/s40814-021-00770-x).	Thank you. We have added a sample size justification to the 'Participants' section.<br>Line 174 *et seq* now reads:<br>A formal sample size calculation was not undertaken as this was a non-randomised feasibility study [30]; our sample size was justified, using two process criteria [46]. From experience with other ADRe studies, we expected a patient participation rate above 60%, and 60-100% to report a potential ADR that could be ameliorated [9, 16-21]. To ascertain a prevalence within 95% confidence intervals of 60% to 100% 15 participants were needed [47].
3. line 33 in the discussion - the authors refer to 'Like most trialists..' - I don't think it is appropriate to generalise in this way so would omit this.	Thank you. The phrase has been removed.
